# Nervous Diseases

**Published:** 1906-06-02

**Authors:** 


### Progress in Medicine and Surgery.

### NERVOUS DISEASES.

**Tumours of the Brain and Cerebral Function.**—Tumours of the brain always form an interesting study, and for various reasons. Such tumours lead, as a rule, to marked and distressing physical and mental change, and tend towards death. It is, therefore, very desirable that by careful study of clinical symptoms and post-mortem records, more light may be thrown on their causation, situation, and amenability to operatic treatment. From the physiologist's point of view, also, much can be learnt in this way as to the localisation of cerebral function, and perhaps in no other department of medicine is it so necessary for the physiologist, pathologist, and clinical observer to work hand in hand. Localisation of cerebral function, especially from the histological standpoint, forms the subject of an important monograph recently published by A. W. Campbell, pathologist to the Asylums Board of the County of Lancaster. In a recent review [1] of this work the main features of the book are summarised. An immense amount of labour has been expended in section cutting, staining, and microscopical examination of human and comparative brains, normal and pathological, and many plates and illustrated descriptions furnish a guide to the histological structure of all parts of the brain. Much use has been made of the valuable experiments of Sherrington and Grünbaum on the anthropoid apes, and although no striking new facts are presented as to the localising of function, the investigations should be found very helpful in advancing the subject. Stewart and Holmes [2] have made a special study of tumours of the cerebellum, both intra- and extra-cerebellar. The latter term includes tumours lying in the angle between the pons and the cerebellum, compressing both, but not directly involving either. The authors consider that "cerebellar tumours rarely, perhaps never, run a latent course, or fail to produce clinical symptoms"; that "this typical symptomatology is never met with in other conditions unassociated with other signs," and that, therefore, "the accurate diagnosis and localisation of the majority of uncomplicated cerebellar neoplasms is possible." In 22 of their 40 cases studied, the diagnosis was confirmed by operation or autopsy. They give a synopsis of the chief symptoms met with in both forms of cerebellar tumour.

*A. Intra-cerebellar Tumours.*—The classical trio of symptoms, headache, vomiting, and optic neuritis, are very early, constant, and pronounced. Headache is most intense, and may be limited to the occipital region, but may also be frontal or retro-ocular. Vertigo is very constant, with a tendency to rotation towards the sound side. Lesions of cranial nerves besides the ocular are but slight. Nystagmus is nearly invariable, and is most marked on looking towards the lesion. Of motor signs, ataxia, atonia, paresis of limbs on the side of the lesion are, in the order named, the essential signs. Ataxia effects the arm more than the leg, and is most marked on the damaged side. It appears only on active movement, and is not increased by closing the eyes. Ataxy of the trunk muscles is held to be largely responsible for the typical " cerebellar gait." Lordosis in the dorso-lumbar region is frequent. Atonia is shown by the flabbiness of the muscles on examination and passive movement, and is not infrequently associated with increased tendon reflexes. But the last named are strikingly variable, though more commonly the deep reflexes are diminished on the side of the lesion. Ankle-clonus does not occur, nor, in uncomplicated cases, is the extensor form of plantar reflex observed. Sensory symptoms are absent, and there are no special mental symptoms, although increased intracranial pressure may cause drowsiness or stupor. The occipital region is often tender.

*B. Extra-cerebellar Tumours.*—Any or all of the general symptoms may occur late in the case, and be poorly marked. Headache is almost constantly occipital, and may radiate down the neck. Vertigo occurs with marked frequency, but differs from that noticed with tumours involving the cerebellum—that is to say, rotation is towards, not away from, the side of the lesion. Cranial nerve lesions are always present, and often marked. The auditory nerve is especially liable to involvement and tinnitus and complete deafness on the side of the tumour occur early. The sensory root of the trigeminus is also often affected, and pain, paræsthesia or anæsthesia and lack of taste on the same side result. Facial paralysis or irritation is also common. Of motor symptoms ataxia similar to that seen in the intra-cerebellar lesion is present, but the gait may be more spastic. This tendency to spasm is more marked in the leg of the sound side, for loss of muscular tone in this variety of cerebellar tumour is rare; rather is there a tendency to hypertonicity and spastic paralysis of the limbs on the opposite side. The reflexes are rarely diminshed, often exaggerated. Ankle-clonus is frequent, and an extensor plantar response may sometimes be obtained. Sensory symptoms are not present apart from the involvement of the trigeminus already mentioned. Although less amenable to operative removal than those tumours which are situated within the cerebellum, these extra-cerebellar tumours have in some instances been successfully removed. The safest and shortest route for getting at them, as pointed out by C. H. Frazier,[3] is along a line parallel to the petrous portion of the temporal bone, so that they are approached from the lateral rather than from the superior or inferior aspect of the cerebellum.

False localising signs of intracranial tumour are very apt to occur from the ventricular distension, w[illegible]ch may arise secondary to a growth in any situation of the brain; but the important point to note about these signs is, as Collier has pointed out, that they occur late in the course of the illness. Collier is in accord with the views expressed by Grainger Stewart, and Holmes when he says that " it appears that lesions of the cerebellum are almost always early productive of characteristic symptoms," so that a tumour giving rise to marked general but very few special symptoms for many weeks may with much certainty be located above the tentorium. Some of the general symptoms of intracranial tumour are discussed by J. Taylor.[4] Headache and vomiting are nearly always associated in the same patient. Amblyopia is one of the signs sometimes associated with optic neuritis, the patients complaining that a blinding mist seems to come over their eyes. It is to be regarded as a sign of a high grade of neuritis. Does, Dr. Taylor asks, the intensity of the optic neuritis give any indication of the probable situation of the tumour? The answer is that " as a class," but with occasional exceptions, neuritis is much more intense in subtentorial tumours than in those involving the hemispheres. As regards difference of intensity on the two sides, this writer thinks that in tumours in the Rolandic and frontal areas neuritis is more likely to be intense on the same side as the lesion, while the opposite holds good of tumours in the occipital and cerebellar regions. Tremor appears to be specially associated with mesencephalic tumours. The occurrence of epileptic fits in a previously healthy adult should be regarded with suspicion lest they should really be one of the early signs of new growth. Some very interesting and instructive cases of cerebral tumour have recently been recorded by Gowers,[5] who describes a remarkable case of multiple periosteal growths involving the cranium and other bones, and also the lungs and alimentary canal. Gardiner and Carey [6] also record cases.

[1] Brit. Med. Jour., Jan. 13, p. 84. [2] Scottish Jour. Med., Feb., p. 138. [3] Ibid., p. 143. [4] Brit. Med. Jour., Dec. 2. [5] Lancet, Dec. 2. [6] Jour. Nerv. and Ment. Dis., Jan.

